# Induction Chemotherapy in Locally Advanced Pharyngolaryngeal Cancers with Stridor: Is It Feasible and Safe?

**DOI:** 10.1155/2012/549170

**Published:** 2012-08-12

**Authors:** Vijay Maruti Patil, Vanita Noronha, Amit Joshi, Vamshi Muddu, Bhavesh Poladia, Bharat Chauhan, Kumar Prabhash, Devendra Arvind Chaukar, Pankaj Chatturvedi, Gouri Pantvaidya, Shashikant Juvekar, Anil D'cruz

**Affiliations:** ^1^Department of Medical Oncology, Tata Memorial Hospital, Mumbai 400012, India; ^2^Department of Surgical Oncology, Tata Memorial Hospital, Mumbai 400012, India; ^3^Department of Radio-Diagnosis, Tata Memorial Hospital, Mumbai 400012, India

## Abstract

*Background*. The standard initial management of patients with locally advanced pharyngolaryngeal presenting with stridor is tracheostomy. Tracheostomy has been shown to negatively impact cancer-related outcomes. *Methods*. Retrospective analysis of prospectively collected data of 9 patients, who underwent induction chemotherapy with the aim of prevention of tracheostomy. Presenting features, time to resolution of stridor, and further management are reported. *Results*. Eight out of 9 patient received chemotherapy within 12 hours of presentation with stridor. There were 4 patients each with primary hypopharynx and larynx. The stage was IVA in 6 patients and IVB in 2 patients. In all patients receiving immediate chemotherapy, clinical stridor resolved within 48 hours. The radiological response rate was 62.5%. The median reduction in size of tumor was 37%. *Conclusion*. Immediate neoadjuvant chemotherapy is a feasible and safe option for patients presenting with early stridor and helps in resolution of stridor and avoiding tracheostomy.

## 1. Introduction

Patients with locally advanced pharyngolaryngeal cancer have a poorer prognosis than patients with localised disease at the same site. Around 10% of these patients may present with symptoms and signs of impending airway obstruction in the form of stridor, which should be managed as an oncological emergency [1]. The standard initial management of patients presenting with stridor is endotracheal intubation, tracheostomy, or laser excision for immediate relief of airway obstruction. Among these, tracheostomy and an artificial airway which bypasses the tumour mass are the most commonly utilised method [2].

However, when patients with tracheostomy are subsequently treated with definitive chemoradiation, the response rate, progression-free survival (PFS), and overall survival (OS) have been reported to be significantly lower, compared to those patients with similar stage of disease who did not undergo tracheostomy [3]. The local control rate is poorer even when the patients undergo laryngectomy. Tracheostomy has been associated with inferior duration of OS and increased rate of distant metastasis in some series [4–6]. 

In the last decade there has been a rapid evolution in the use of induction chemotherapy for locally advanced head and neck cancers. The established benefits of induction chemotherapy include a decrease in tumor size and in vivo identification of tumor sensitivity to chemoradiation. Induction chemotherapy regimens containing taxanes and platinum compounds are associated with response rate around 60–80% in advanced laryngeal and hypopharyngeal squamous cell cancers [7–11].

We hypothesised that patients with advanced laryngopharyngeal cancers who present with stridor might have a decrease in tumour bulk with immediate induction chemotherapy. Such an approach could potentially avoid respiratory compromise and the need for an emergency tracheostomy. The aim of the present study was to evaluate the efficacy and safety of using immediate induction chemotherapy to avoid tracheostomy in patients with early stridor.

## 2. Materials and Methods

We performed a retrospective analysis of prospectively collected data of patients who had locally advanced pharyngolaryngeal squamous cell carcinoma, presented with early stridor (CTCAE version 4.02 Grade 3), and were treated with induction chemotherapy with the aims of reducing tumour bulk and avoiding tracheostomy. 

After discussing the treatment options with the patients and caregivers, including the potential benefits and possibility of future organ preservation, the patients who gave informed consent for this approach were admitted for planned treatment. 

The patients willing for chemotherapy were treated with doublet or triplet chemotherapy regimens containing platinum compounds and taxanes with or without 5-fluorouracil. The choice of platinum was determined by the serum creatinine clearance; patients with serum creatinine clearance below 60 mL/min were treated with carboplatin and those with creatinine clearance above 60 mL/min were treated with cisplatinum. Standard premedications and adequate hydration were ensured in all patients. The doses of chemotherapy and drug schedule are shown in Table 1.

The initial chemotherapy was given under strict medical supervision. Patients were clinically monitored by two senior team members belonging to the departments of medical oncology and surgical oncology for resolution of respiratory compromise and impending obstruction. Pulse rate, respiratory rate, blood pressure, and oxygen saturation by means of noninvasive pulse oximetry were recorded continuously. Equipment and operating theatres needed for emergency tracheostomy were kept ready in case the patient had progressive respiratory compromise during chemotherapy. The time taken for symptomatic relief of respiratory distress and resolution of stridor was recorded.

Patients who had resolution of stridor were observed for at least 5 days in the hospital during the chemotherapy and were later given one more cycle of chemotherapy. Grade 3-4 toxicity during chemotherapy was recorded in accordance with CTCAE version 4.02. The patients underwent a radiological response assessment after 2 cycles of chemotherapy with axial imaging and then were planned for local treatment in a multimodality joint clinic consisting of surgical oncologists, radiation oncologists, and medical oncologists. The intent was for organ preservation whenever feasible. The treatment plan and reasons for adopting either approach were documented. 

Statistical analysis was done with SPSS version 16. We defined “stridor prevention (SP)” as successful control of stridor within 2 days of administration of chemotherapy and absence of recurrence of stridor till 4 weeks after completion of last dose of chemotherapy.

## 3. Results

A total of 9 patients of locally advanced pharyngolaryngeal cancers presented with early stridor during December 2010 to June 2011. Eight patients willing for treatment received immediate chemotherapy (within 12 hours of presentation to the hospital) under stringent clinical monitoring. The baseline characteristics of these patients are shown in Table 2. All patients had locally advanced disease with the stage being stage III or IV. The median BMI was 20.5 Kg/m^2^. Median albumin was 3.7 g/dL and the median haemoglobin was 11.75 g/dL. All patients had eastern cooperative oncology group (ECOG) performance status (PS) of 3, primarily due to impending respiratory compromise and resultant stridor.

One patient was unwilling for any treatment, either immediate chemotherapy or tracheostomy. This patient presented later to the emergency medical services with grade 4 stridor and had to be relieved of airway obstruction by a tracheostomy. The details of the other patients are listed in Table 2.

The details of the chemotherapy received are shown in Table 3. Six patients received doublet and 2 patients were given 3-drug regimen. Docetaxel and paclitaxel were used in 5 and 3 patients, respectively. All the patients had complete clinical resolution of stridor within 48 hours. There was no respiratory distress on subsequent followup. One patient (patient number 6) defaulted after the first cycle and did not receive any further treatment at our institute. Another patient (patient number 3) received only one cycle of chemotherapy as patient had logistic issues in continuing treatment at our centre and wanted further treatment at his native place. The remaining patients received a median of 2 cycles of chemotherapy before assessment. None of the patients had complete response, 5 had partial response, and 2 had stable disease on axial imaging. The median reduction in tumor size was 37%. Based on the discussion in the multimodality clinic, all the patients could be treated with intent of organ preservation. All the patients except one were given external beam radiotherapy concurrent with weekly cisplatin. That one patient received only external radiation only. All except one patient have completed their local treatment and are on regular followup. The follow-up periods are insufficient to comment on survival outcomes. 

The initial aim of stridor prevention was achieved in all patients. All the patients had complete clinical resolution of stridor within 48 hours, the earliest being at 24 hours. The earliest in 24 hours of start of induction chemotherapy. There were no episodes of recurrence of stridor in between or just prior to the start of subsequent chemotherapy cycle.

## 4. Discussion

Patients with locally advanced pharyngolaryngeal cancer presenting with stridor (grade 3 or 4) routinely undergo tracheostomy as an immediate procedure [4]. These patients are generally not considered for organ preservation in view of their large tumor bulk and undergo laryngectomy, whenever feasible. This approach has important implications with respect to the eventual outcome. Chernov et al. showed that patients, who underwent surgery after tracheostomy, had higher rates of local recurrence and distant metastasis, leading to a poorer overall survival [5]. The adverse impact of tracheostomy on overall survival has also been demonstrated in a retrospective analysis by Herchenhorn et al. in patients treated with chemoradiation for laryngeal carcinoma. Shorter progression-free survival (HR 2.83, CI 95% 1.60–4.88, *P* < 0.001) and median overall survival (12 versus 56 months, HR 2.37, CI 95% 1.43–3.93, *P* < 0.001) were seen in patients who had undergone tracheostomy. The impact of previous tracheotomy was not altered when adjusted by other prognostic factors (HR 8.7, CI 95% 3.1–24.0, *P* < 0.001) [3]. Similar findings have been observed in T3 transglottic carcinomas and, in one series, the patients with tracheostomy had a 5-year OS of 20% as opposed to 80% in those patients with T3 transglottic tumor without tracheostomy. This study is important as it dealt with only T3 transglottic carcinoma. Further all patients underwent total laryngectomy. So in this study both the T stage and treatment were similar in both subgroups of patients with and without tracheostomy. Trachesostomy was again showed to be a independent important prognostic variable in a multivariate analysis [6].

Whether tracheostomy itself is an independent negative prognostic factor or a marker for tumors with large volume and advanced stage leading to a poor outcome is uncertain. There are conflicting reports in the literature with no consensus on the issue [1, 3–6, 12–14]. However in one study reported by Menedhall et al. in patients with T3 transglottic carcinoma the tumor bulk was taken into account by CT volumetric estimation, yet pretreatment tracheostomy was significantly related to diminished cause-specific survival (*P* = 0.0345). The results from the above-mentioned studies [3–6] allow us with reasonable confidence to assume that pretreatment tracheostomy is associated with poor outcome and efforts to prevent it if possible are required.

Induction chemotherapy might however be beneficial in both scenarios, by helping avoid tracheostomy and by overcoming the negative prognostic implication of tracheostomy if they exist and in cases of bulky tumors by medical debulking of large local primaries, identifying patients likely to respond to radiation and improving their local controls [9–11]. 

According to the common terminology criteria for adverse effects (CTCAE version 4.02), stridor is graded clinically. Grade 3 stridor denotes respiratory distress limiting self-care and activities of daily living which requires therapeutic medical intervention. Grade 3 stridor can be managed medically. However this classification is subjective. Kamath et al. have given objective classification of stridor as mild, moderate, and severe on the basis of arterial blood gas values [15]. This classification might be a more objective criterion for determining the selection of patients for initial induction chemotherapy. We would advocate efforts of avoidance of tracheostomy by induction chemotherapy in grade 3 stridors by CTCAE 4.02 or mild stridor by Kamath et al. classification.

Steroids are generally recommended as an initial medical management for stridor. Though all the patients in our cohort received steroids (dexamethasone 16 mg intravenous) as part of the premedication before chemotherapy, we do not believe this single dose of steroid had a prolonged or significant effect in decreasing stridor. While there could be immediate symptomatic relief, steroids alone cannot result in complete sustained resolution of stridor which was seen in our patients with chemotherapy. 

The present report is a small case series of only 8 patients and is insufficient to comment on the effectiveness of different chemotherapy regimens used. We have used both 2- and 3-drug regimens containing platinum and taxane with or without 5-fluorouracil. However in view of better response rates being documented in multiple randomized trials for induction chemotherapy, the utilization of 3-drug regimen would probably be a more evidence-based approach [16, 17].

Induction chemotherapy has been evaluated in large randomised, controlled trials and has shown benefit in terms of response rates, progression-free and overall survival. These trials were done for both unresectable and operable tumours [16–18]. The use of induction chemotherapy in our patients highlights the fact that these tumors are often chemoresponsive and that clinical response occurs relatively early. Though no immediate axial imaging or invasive examination was done in our patients, resolution of stridor can be taken as an evidence of early response. Also doing a indirect or direct laryngoscopic examination was not considered safe and feasible in patients in whom stridor has just been relieved. Stridor is produced by phonation produced by ingress of air across a narrow airway during inspiration. Subsequently, this phonation would decrease or stop when the lumen would be cleared of the obstruction or mass [19]. In our patients, the clearance of tumour by chemotherapy resulted in clinical resolution of stridor. 

In patients presenting with early stridor (CTCAE grade 3), chemotherapy can be safely administered under close monitoring and watchful waiting with a provision for urgent surgical intervention. Though followup is required to prove the long-term effects of using induction chemotherapy, especially the rates of locoregional relapse, we believe that this approach helps in avoiding tracheostomy and achieving organ preservation. Further studies with larger sample size and in multiple centres would be required to prove or disprove the validity of this approach in overcoming the negative prognostic impact of tracheostomy or tumor bulk in these patients.

## 5. Conclusion

In conclusion, we believe that induction chemotherapy is a novel and effective treatment for patients of locally advanced pharyngolaryngeal cancers presenting with early, non-life-threatening stridor which could potentially avoid tracheostomy and help in organ preservation. However this should not be considered as a standard practice such treatments could offer in control settings as described in the methodology.

## Figures and Tables

**Table 1:** Doses given in the regimens. In 2-drug regimen either paclitaxel or docetaxel was used and the choice of platinum was based on the serum creatinine clearance.

*3-drug regimen (DCF)*	* *
Docetaxel	75 mg/m^2^ on D1
Cisplatin	75 mg/m^2^ on D1
5-FU	750 mg/m^2^ from D1–D5
*2-drug regimen*	
Paclitaxel (P) or docetaxel (D)	P—175 mg/m^2^ on D1 or D—75 mg/m^2^ on D1
Carboplatin (carbo) or cisplatin (cis)	Carboplatin (AUC 5) or cisplatin 75 mg/m^2^

**Table 2:** Baseline characteristics.

Patient	1	2	3	4	5	6	7	8
Age in years	53	45	45	42	71	47	66	55
Sex	M	F	M	F	M	F	M	M
Comorbidity	none	none	none	none	IHD	none	none	none
Primary site	Supra glottis	PFS	Post cricoid	PFS	Supra glottis	PFS	Supra glottis	Supra glottis
T stage	3	4a	3	4a	3	3	3	4
Minor thyroid cartilage invasion	No	Yes	No	Yes	No	No	No	yes
N stage	0	0	3	1	2c	2c	0	2a
Stage	III	IVa	IVb	IVa	IVa	IVa	III	IVa
BMI (Kg/m^2^)	22	15	27	18	24	18	19	22
ECOG performance status	3	3	3	3	3	3	3	3
Haemoglobin (g/dL)	10	9.6	12	11.5	13	11	12	13
Albumin (mg/dL)	4	3	4	4.2	3.7	3.8	3.6	3.7

IHD: ischemic heart disease, PFS: pyriform sinus, M: male, F: female.

**Table 3:** Treatment details.

Patient	1	2	3	4	5	6	7	8
Regimen	D + C	D + carbo	P + carbo	P + carbo	P + carbo	DCF	DCF	D + C
Time for complete resolution of stridor (in hours)	24	24	48	48	24	24	24	24
Number of cycles	3	2	1	2	2	1	3	2
Response	SD	PR	PR	SD	PR	Not assessed	PR	PR
Grade 3-4 haematological toxicity	Nil	Yes	Nil	Nil	Nil	Nil	Yes	Yes
Grade 3-4 Gastrointestinal toxicity	Yes	Nil	Nil	Nil	Nil	Nil	Yes	Nil
% reduction in tumor size	10	47	37	20	40	Not assessed	80	37
Local Rx subsequently offered	CRT	CRT	RT	CRT	CRT	Defaulted	CRT	CRT

D: docetaxel, C: cisplatin, Pacli: paclitaxel, Carb: carboplatin, F: 5FU, CR: complete response, PR: partial response, SD: stable response, CRT: chemoradiation and RT: radical radiation.
